# The Sodium and Potassium Content of the Most Commonly Available Street Foods in Tajikistan and Kyrgyzstan in the Context of the FEEDCities Project

**DOI:** 10.3390/nu10010098

**Published:** 2018-01-16

**Authors:** Inês Lança de Morais, Nuno Lunet, Gabriela Albuquerque, Marcello Gelormini, Susana Casal, Albertino Damasceno, Olívia Pinho, Pedro Moreira, Jo Jewell, João Breda, Patrícia Padrão

**Affiliations:** 1 Institute of Tropical Medicine and International Health, Charité-Universitätsmedizin Berlin, Campus Virchow-Klinikum, Augustenburger Platz 1, 13353 Berlin, Germany; 2 Division of Noncommunicable Diseases and Life-Course, World Health Organization (WHO) Regional Office for Europe, UN-City, Marmorvej 51, DK-2100 Copenhagen Ø, Denmark; marcello.gelormini@gmail.com (M.G.); jewellj@who.int (J.J.); rodriguesdasilvabred@who.int (J.B.); 3 EPIUnit—Instituto de Saúde Pública, Universidade do Porto, Rua das Taipas nº 135, 4050-600 Porto, Portugal; nlunet@med.up.pt (N.L.); gabriela.albuquerque@ispup.up.pt (G.A.); sucasal@ff.up.pt (S.C.); pedromoreira@fcna.up.pt (P.M.); patriciapadrao@fcna.up.pt (P.P.); 4 Departamento de Ciências da Saúde Pública e Forenses e Educação Médica, Faculdade de Medicina da Universidade do Porto, Alameda Prof. Hernâni Monteiro, 4200-319 Porto, Portugal; 5 Faculdade de Farmácia, Universidade do Porto, Rua Jorge de Viterbo Ferreira 228, 4050-313 Porto, Portugal; 6 REQUIMTE, Laboratório de Bromatologia e Hidrologia, Universidade do Porto, Rua Jorge de Viterbo Ferreira 228, 4050-313 Porto, Portugal; oliviapinho@fcna.up.pt; 7 Faculdade de Medicina da Universidade Eduardo Mondlane, Avenida Salvador Allende nº 702, 257 Maputo, Moçambique; tino_7117@hotmail.com; 8 Faculdade de Ciências da Nutrição e Alimentação da Universidade do Porto, Rua Dr. Roberto Frias, 4200-465 Porto, Portugal; 9 Centro de Investigação em Atividade Física, Saúde e Lazer, Universidade do Porto, Rua Dr. Plácido da Costa, 4200-450 Porto, Portugal

**Keywords:** sodium, potassium, sodium–potassium ratio, ready-to-eat food, street food, Tajikistan, Kyrgyzstan, low- and middle-income countries

## Abstract

This cross-sectional study is aimed at assessing sodium (Na) and potassium (K) content and the molar Na:K ratios of the most commonly available ready-to-eat street foods in Tajikistan and Kyrgyzstan. Four different samples of each of these foods were collected and 62 food categories were evaluated through bromatological analysis. Flame photometry was used to quantify sodium and potassium concentrations. The results show that home-made foods can be important sources of sodium. In particular, main dishes and sandwiches, respectively, contain more than 1400 and nearly 1000 mg Na in an average serving and provide approximately 70% and 50% of the maximum daily recommended values. Wide ranges of sodium content were found between individual samples of the same home-made food collected from different vending sites from both countries. In industrial foods, sodium contents ranged from 1 to 1511 mg/serving in Tajikistan, and from 19 to 658 mg/serving in Kyrgyzstan. Most Na:K ratios exceeded the recommended level of 1.0 and the highest ratios were found in home-made snacks (21.2) from Tajikistan and industrial beverages (16.4) from Kyrgyzstan. These findings not only improve data on the nutritional composition of foods in these countries, but may also serve as baseline information for future policies and interventions.

## 1. Introduction

Non-communicable diseases (NCDs) are the leading cause of death globally, accounting for 70% of the total estimated deaths in 2015 [1]. In the World Health Organization (WHO) European Region, NCDs alone are responsible for 89% of total deaths [1]. Approximately 80% of these deaths occur in low- and middle-income countries (LMICs) [2] and the situation is of particular concern in Newly Independent States (NIS) such as Tajikistan and Kyrgyzstan, where 57% and 47%, respectively, of all NCD-related deaths occur prematurely [3].

This epidemiological transition observed in LMICs (including the NIS) [4], has been accompanied by a nutrition transition with conspicuous changes in the supply, availability, and consumption of foods [5,6,7,8]. Dietary patterns are rapidly shifting towards a greater consumption of animal-source foods, refined grains, and processed foods, frequently high in saturated fat, *trans* fatty acids, free sugars, and/or salt [7,9], as well as lower consumption of fruits and vegetables [7,10]. Specifically, diets high in sodium (Na) and low in potassium (K) have long been identified as important drivers for NCD-related disability and mortality [11].

Sodium intake is closely associated with blood pressure (BP) [12,13] and high BP itself is an important risk factor for NCDs, particularly cardiovascular diseases (CVDs). [11]. The relationship between sodium intake and CVD morbidity and mortality is suggested to vary according to different sodium intake levels, following a J-shaped curve: low intakes (less than 2 g/day) show an inverse association with CVD outcomes; excessive intakes, particularly those above 4 g/day of sodium, show a direct impact; and intermediate intakes do not appear to have measurable effects [14]. The WHO recommends a maximum daily intake of 2 g of sodium per day [15].

Additionally, low potassium intake has been associated with increased risk of high BP and stroke, while an intake of 90 mmol/day (≈3510 mg) is potentially protective against these conditions [16,17,18]. The role of potassium in diminishing the effect of high sodium intake in BP has also been established [19]. Furthermore, sodium-to-potassium (Na:K) ratios were shown to be key predictors of BP and CVD outcomes [20,21,22,23] and ratios close to 1.0 are considered beneficial for health [24]. As such, the WHO recommends that countries implement salt reduction strategies and promote increased consumption of fruit and vegetables, among other policies, to improve diets in the region [25].

New urban living patterns are marked by less time for home food preparation and increased consumption of food prepared away from home, notably street food [26,27]. Street food provides an accessible and affordable source of nutrition for many people living in LMICs. Nevertheless, little is known about the nutritional contribution of these foods to the diet, despite the high amounts of sodium that might be expected in many food products sold by street vendors [28].

This study is set in the context of the WHO project FEEDCities, which is a multi-country project in Central Asia, the Caucasus, and south-eastern Europe that aims to fill the gap of lack of information available on the nutritional composition of the available ready-to-eat street food in the WHO European Region [29,30]. The main objectives of this project are to characterize street food environments in urban contexts, to document the types of foods most commonly available on the streets, and to assess their nutritional composition. This paper will exclusively focus on the latter objective, in particular, on the analysis of sodium and potassium content of street foods (including beverages) sold in urban areas of Tajikistan and Kyrgyzstan. We aim to describe the variability across different types of foods and similar food products acquired in different vending places to illustrate the potential for improvement of street food availability and use.

## 2. Materials and Methods

The present study is a cross-sectional evaluation of the sodium and potassium content and the Na:K ratio of the most commonly available street foods collected from the streets of Dushanbe and Bishkek, the capital cities of Tajikistan and Kyrgyzstan, respectively. After approval (reference number CE16058) from the Ethics Committee of the Institute of Public Health of the University of Porto (ISPUP) and local authorities, the study was carried out between April and May 2016 in Dushanbe, and between June and July 2016 in Bishkek.

The study adopted the definition of street food proposed by the Food and Agricultural Organization (FAO) and the WHO of “ready-to-eat foods and beverages prepared and/or sold by vendors or hawkers especially in the streets and other similar places” [31]. This includes prepared or cooked foods to be consumed immediately or later on (e.g., at work), without any further preparation needed.

Through a first exploratory visit to the cities, it was observed that the street food vending was typically occurring in and around bazaars and public markets (hereinafter “markets”). A comprehensive list of these venues, including a total of 36 markets in Dushanbe and 19 in Bishkek, was provided by the local authorities. From each city, 10 markets were randomly selected, including small to large-sized markets, among those selling only food or food and other goods. In Dushanbe, the final list ensured a representation of all four districts of the city proportional to the corresponding number of markets. In Bishkek, the final list represented three out of the four districts.

The study area was defined by selecting a 500-m-diameter buffer for each selected market, with the centroid in the geographic midpoint of the market, which covered the market and its surroundings. Preliminary data on the street food on offer was collected from vending sites operating within this study area. Eligible vending sites comprised those selling ready-to-eat food from any venue, including both fixed and mobile vending units, selling directly on the street. Street hawkers were also covered. Vending sites selling exclusively fresh or dry fruits in natura were not eligible. In Tajikistan, all eligible vending sites within the study area were assessed, whereas in Kyrgyzstan, taking into account that the selected markets in Bishkek had a larger number of eligible vending sites, of every two vending sites, the second was systematically evaluated.

In the first phase of the study, 10 trained field researchers gathered preliminary data on the availability of the ready-to-eat food, mostly by direct observation. Data was collected through mobile phones. The existing android-based tool ‘KoBoCollect’ [32], by ‘KoBoToolbox’, was used to administer an electronic questionnaire, containing both closed- and open-ended questions that helped researchers record the characteristics of the food products sold. Operating in pairs, the researchers canvassed each selected market and all publicly accessible streets in their surroundings within predefined study areas. For the eligible vending places identified, the researchers registered the corresponding position through Global Positioning System (GPS) coordinates, and described the type of foods being sold as well specific serving sizes, and took photographs of the portions typically served. Data collection took place during weekdays and weekends to ensure the evaluation of the food on offer was representative.

Foods available were grouped into two broad types according to their elaboration and degree of processing [33]: (1) home-made food, defined as foods and beverages cooked and/or prepared at home or on the street; and (2) industrial foods, comprising foods and beverages that are industrially produced using industrial techniques.

The most commonly available street foods, both homemade (20 in Bishkek and 25 in Dushanbe) and industrial (10 in each country) were then selected for bromatological analysis by ranking the number of occurrences of each food registered during preliminary data collection on food offer. The larger number of home-made foods to be sampled is explained by the fact that the nutritional composition of these foods is often not known, as opposed to industrial foods. Fruits in natura, water, coffee, tea, and soft and alcoholic drinks were excluded from the sample collection as their nutritional values have been well studied and are not expected to vary significantly in terms of sodium and potassium contents.

### 2.1. Food Sample Collection

The most common home-made and industrial foods identified were split in equal sets of five in each country. For 10 consecutive days, including weekdays and weekends, two sets for home-made foods and two sets for industrial foods were collected in each of the 10 study areas of Dushanbe and Bishkek, until four samples of each of the most common foods in each country were collected from four different vending sites. However, for some foods (from Tajikistan), it was only possible to collect three samples. A total of 254 food samples was collected.

The collection started in the study areas with the least number of vending sites registered during data collection. In each market and its surroundings, 10 GPS coordinates—corresponding to the location of the 10 previously assessed vending sites—were randomly selected for purchasing the corresponding sets for the day. In each market and for each day, only one sample of homemade and/or one sample of industrial food was obtained from the same vending site. In the case it was not possible to buy foods in the selected coordinates, a systematic selection procedure (north, clockwise) was followed until reaching vending sites where the selected foods were available.

Samples were purchased as part of regular transactions, and therefore no consent was required. For each item, the amount of food bought corresponded to the usual serving size, according to the typical consumption pattern observed.

All samples were weighed and prepared for analysis; liquids were homogenized and solid foods were triturated with a food grinder. Four portions of each sample were individually packed in small containers and weighed before being stored in freezer at –18 °C until analysis.

### 2.2. Bromatological Analysis

For analysis of the nutritional composition of the foods collected, samples were defrosted and each container weight was compared with the one registered before being frozen, with no significant variations detected. Each container was carefully homogenized for reincorporation of any condensate/leach and immediately analyzed.

Sodium and potassium were evaluated by flame photometry, on duplicate 2-g portions of each sample according to a previously validated method [34]. Sample extracts were diluted to fit the linear range of the photometric response, using both standard and sample controls through the determinations. The caloric values of samples were estimated after proximate analysis of food components (moisture, protein, total fat and ash) performed in accordance with standard methods, as recommended by the FAO, and using the general Atwater values [35]. All the analytical results are the average of at least two determinations, expressed with respect to 100 g of fresh food.

### 2.3. Data Analysis

**Figure 1:**
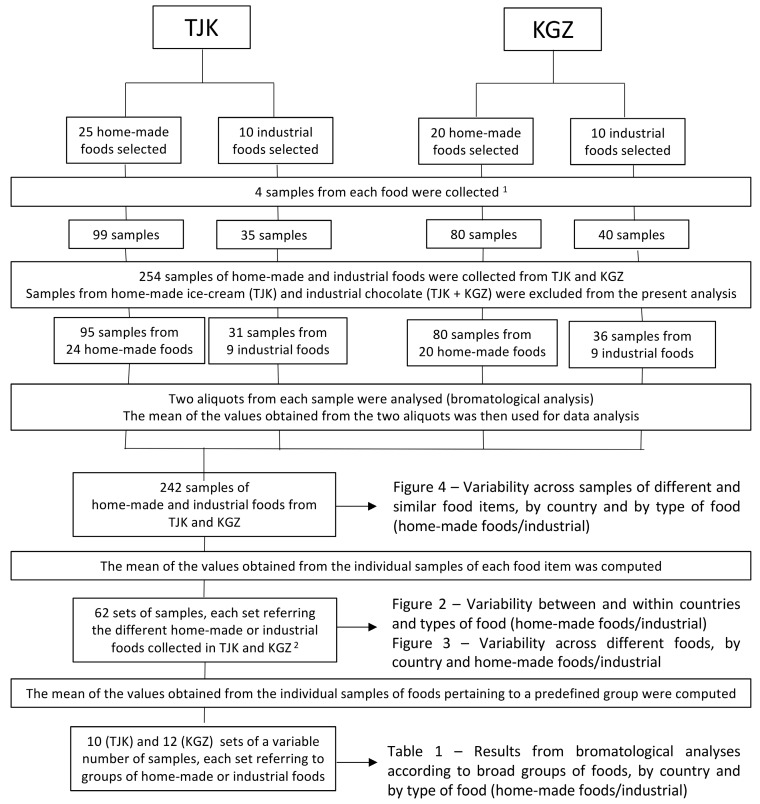
Flow chart of the data analysis process. TJK: Tajikistan; KGZ: Kyrgyzstan. ^1^ Except for home-made sweet pastries and industrial bread, chips, biscuit rolls, dry bread crumbs and wafers, all from Tajikistan, for which only three samples were collected. ^2^ The 62 sets of samples correspond to a total of 46 different foods.

Figure 1 shows a flow chart displaying the data analysis process. From the 254 samples collected, samples of industrial chocolate from both countries, as well as home-made ice-creams from Tajikistan, were omitted from the detailed analysis due to their low-sodium content. Therefore, 242 samples were taken into account, corresponding to the 62 most common foods from both countries.

These 62 foods were further assigned to eight broader groups that were created based on the groups of the WHO nutrient profile model [36] and new ones were extended when necessary: (1) beverages; (2) bread; (3) buns; (4) cakes and cookies; (5) main dishes; (6) sandwiches; (7) savoury pastries and (8) snacks. Based on these groups, a total of 10 (Tajikistan) and 12 (Kyrgyzstan) sets of a variable number of samples were created, each set referring to broader groups of home-made or industrial foods.

Average serving sizes per food, in grams, were calculated as the mean of the individual doses of each of the samples collected for the respective foods. For the food group analysis, the average of serving sizes of the respective food items included in the group was calculated.

Per-serving sodium and potassium levels were expressed in milligrams (mg)/serving. To calculate individual molar Na:K ratios, contents of sodium and potassium (mg/serving) of each sample were converted in millimoles (mmol) using the conversion 23 mg sodium = 1 mmol sodium and 39 mg potassium = 1 mmol potassium.

The average contents of sodium and potassium as well as the molar Na:K ratio of the foods was obtained from the mean of the individual samples collected for the corresponding food [37]. For food group analysis, the averages of sodium, potassium and molar Na:K ratios of the respective foods included in the groups were calculated and the contribution of the sodium and potassium contents of each group to these nutrients’ recommended daily intake was computed.

Sodium and potassium contents of individual samples and foods were further calculated as mg/2000 kilocalories (kcal), taking into account the average daily energy intake in adults.

Statistical analysis was conducted with Stata. Descriptive statistics were used to present results from the nutritional composition of foods. Three box plots were produced to describe the distribution of sodium, potassium, and Na:K ratio/serving, by type of food (home-made vs. industrial) and by country. Mean values of foods were used to describe data of home-made and industrial foods. Scatter plots were obtained with the average sodium and potassium contents, per food, in mg/2000 kcal, and compared with the WHO’s recommended limits [15,18].

A detailed analysis of sodium and potassium contents (mg/2000 kcal) of each food sample was also presented through scatter plots displayed by food groups.

The mean values were obtained from the individual samples of foods for presenting the results according to the predefined groups of foods.

The nonparametric Mann–Whitney U test was used for comparisons between home-made and industrial foods and between countries.

## 3. Results

**Figure 2:**
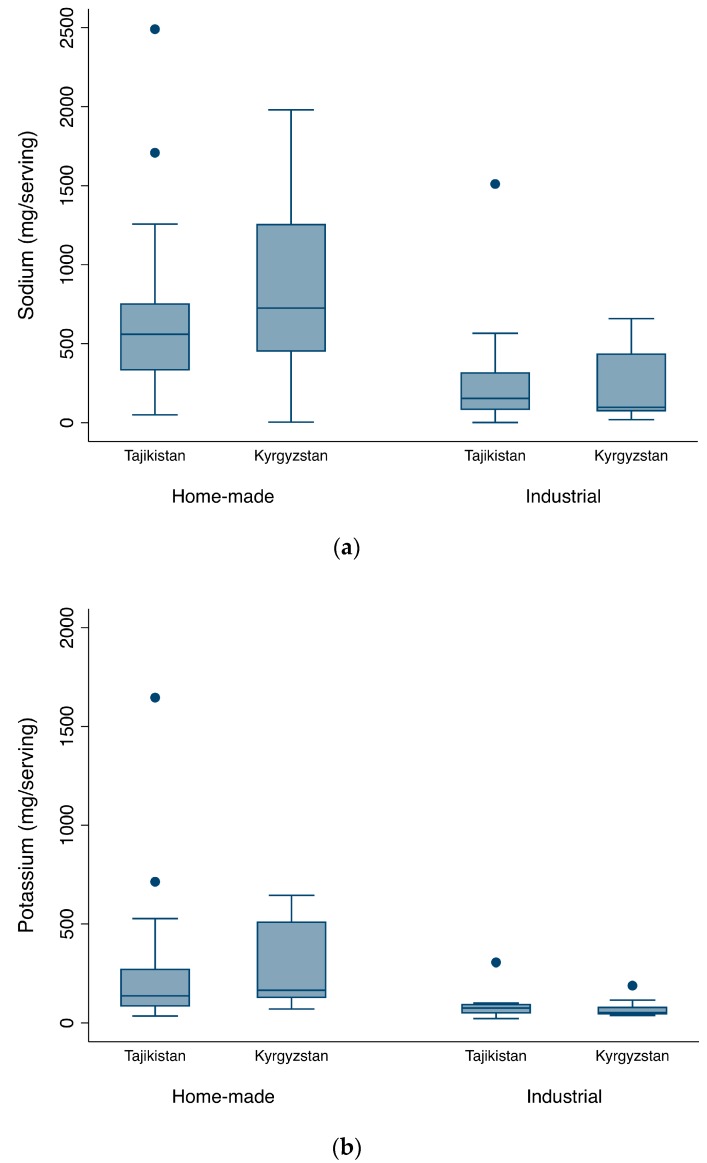

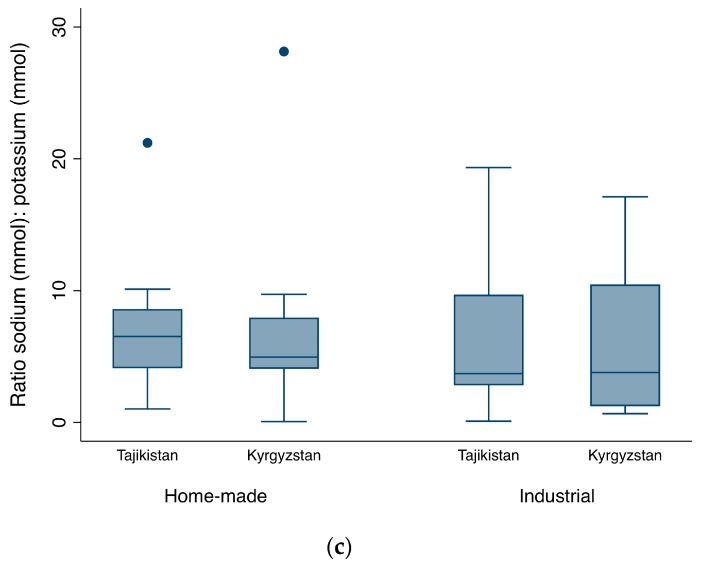
Per-serving average of (**a**) sodium; (**b**) potassium content; and (**c**) molar sodium:potassium ratio of the most commonly available ready-to-eat home-made and industrial foods in Tajikistan and Kyrgyzstan. Outliers are indicated by dots (•).

Figure 2a–c, respectively, show the distribution of the average sodium and potassium contents and the molar Na:K ratios of the 62 most common home-made and industrial foods of Tajikistan and Kyrgyzstan. There were no significant differences between countries with respect to sodium (home-made, *p*-value = 0.211; industrial, *p*-value = 0.691), potassium (home-made, *p*-value = 0.158; industrial, *p*-value = 0.691) or Na:K ratios (home-made, *p*-value = 0.585; industrial, *p*-value = 0.965).

Overall, Figure 2a shows that home-made foods in both countries have higher levels of sodium when compared to industrial foods (Tajikistan, *p*-value = 0.032; Kyrgyzstan, *p*-value = 0.002), as well as greater variability in the average levels of sodium per serving. In Kyrgyzstan, nearly 75% of home-made foods had a content exceeding 500 mg Na/serving, while some of them reached almost 2000 mg Na/serving. In Tajikistan, the median sodium content per serving in home-made foods was 560 mg Na (335–751) although some foods reached 2500 mg of sodium per serving. Regarding sodium content in industrial foods, 75% of foods did not reach 500 mg Na/serving in both countries. However, in Tajikistan, this content ranged from 1 to 1511 mg/serving, while in Kyrgyzstan, sodium content of industrial food was in the range of 19 to 658 mg/serving, with larger dispersion of sodium contents above the median.

Figure 2b shows that potassium contents were also higher in home-made foods of both countries (Tajikistan, *p*-value = 0.019; Kyrgyzstan, *p*-value < 0.001), in comparison to industrial foods. The potassium content of home-made foods ranged between 35 and 1646 mg/serving in Tajikistan and between 78 and 634 mg/serving in Kyrgyzstan. The variability in potassium was much lower in industrial foods and per-serving medians were 75 mg (51–93)/serving, in Tajikistan, and 52 mg (47–79)/serving in Kyrgyzstan.

With respect to molar Na:K ratios, Figure 2c suggests that, on average, industrial foods present higher variability than home-made foods, though differences were not statistically significant (Tajikistan, *p*-value = 0.571; Kyrgyzstan, *p*-value = 0.354). In industrial foods, ratios ranged from 0.1 to 19.3 in Tajikistan, and from 0.7 to 17.1 in Kyrgyzstan. In Tajikistan, the ratios of home-made foods ranged from 1.1 to 10.1 and in Kyrgyzstan from 1.6 to 9.9, reaching up to 21.2 and 28.1, respectively.

**Figure 3:**
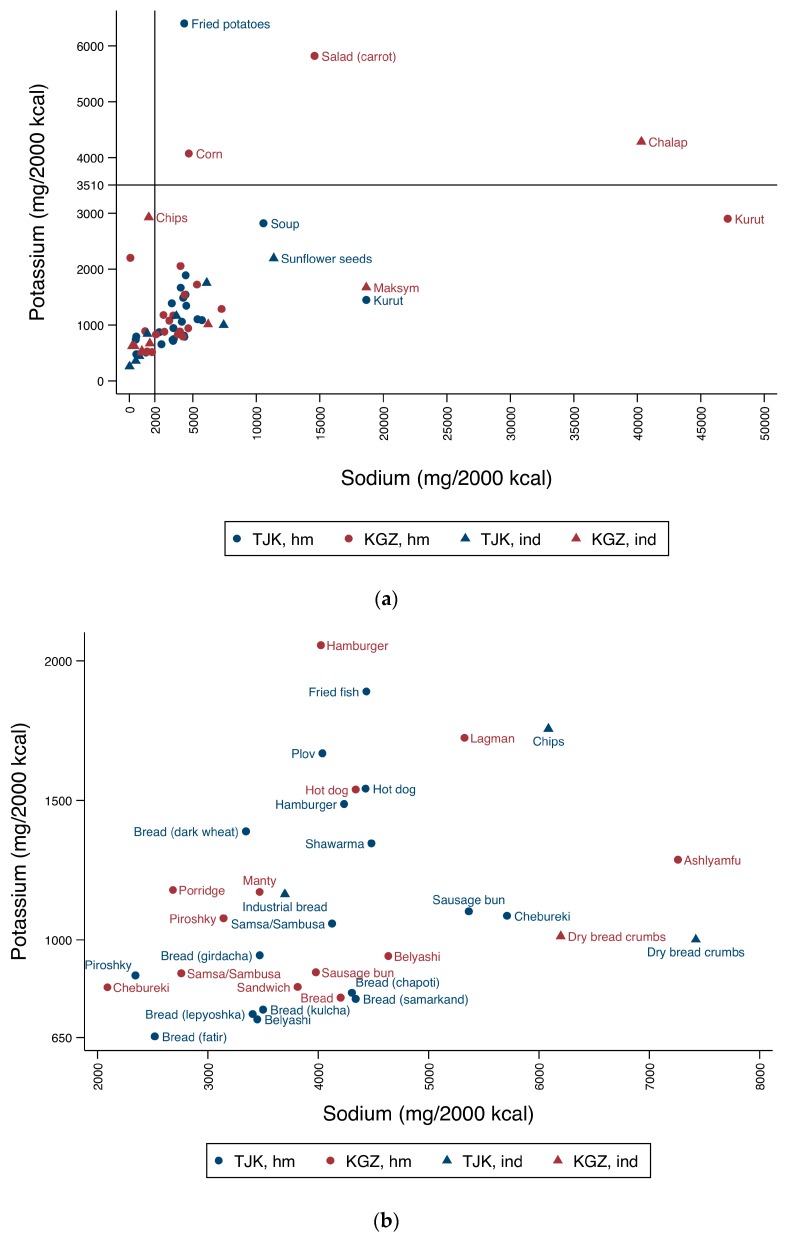

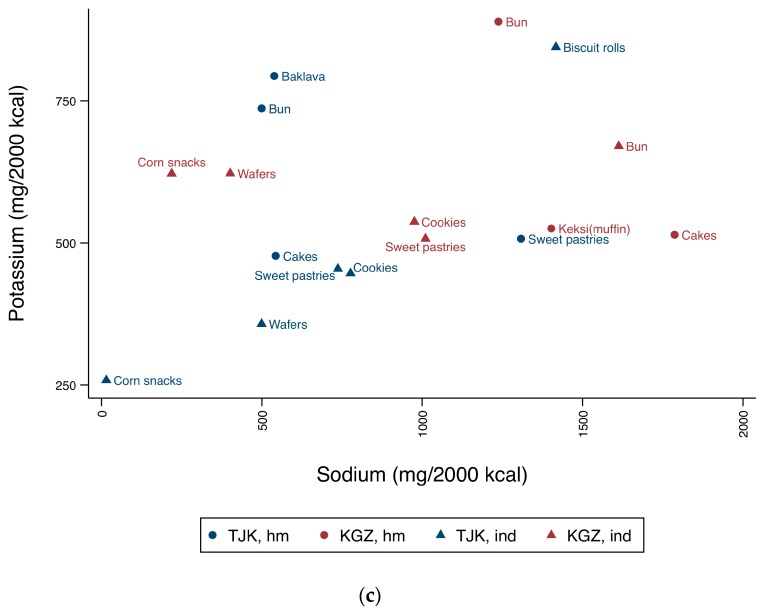
Distribution of average of sodium (Na) and potassium (K) contents (mg/2000 kcal) of the most commonly available home-made (hm) and industrial (ind) foods in Tajikistan (TJK) and Kyrgyzstan (KGZ) relative to the World Health Organization (WHO) sodium and potassium recommendations (less than 2000 and at least 3510 mg/day, respectively); 2000 kcal was assumed as the average energy requirement for adults. (**a**) A general view of these nutrients’ content in all foods collected from both countries, and zoomed-in views of foods that tended to have contents that either (**b**) exceeded sodium upper recommended limits up to 8000 mg Na/2000 kcal and were below minimum potassium recommendations down to 650 mg K/2000 kcal; or (**c**) were below sodium upper recommended limits and below 1000 mg K/2000 kcal.

Figure 3 displays the distribution of average sodium and potassium contents, adjusted to a 2000 kcal diet, in relation to WHO recommended limits for these nutrients [15,18].

In Figure 3a it is possible to identify two well-defined groups of foods (bottom left), where foods from both countries fell short of potassium recommendations and exceeded (Figure 3b) or complied (Figure 3c) with sodium recommended limits. Home-made fried potatoes (Tajikistan), presented the highest average of potassium content—almost twice the minimum daily recommendation. Carrot salad, corn and the industrial drink *chalap* (all from Kyrgyzstan) followed, with average potassium contents between 6000 and 4000 mg K/2000 kcal. The dried milk-based home-made snack *kurut* (Kyrgyzstan) and soups (Tajikistan) presented potassium contents, per 2000 kcal, slightly below the minimum potassium threshold. At the same time, these six foods exceeded maximum daily sodium recommended limits; yet *chalap* had over 20 times more the upper recommended limit for sodium, surpassed only by home-made *kurut* (also from Kyrgyzstan). This was the uppermost average sodium content value of all the foods collected from both countries (47,117 mg Na/2000 kcal). *Kurut* collected in Tajikistan also presented high sodium content, exceeding upper recommended limits by more than nine times. *Maksym*, a Kyrgyz drink usually sold together with *chalap*, and sunflower seeds (Tajikistan) were also high in sodium, with contents close to 20,000 mg Na/2000 kcal. In Kyrgyzstan, industrial chips, had an average sodium content below upper recommended limits and potassium content close to the threshold of 3510 mg K/2000 kcal (Figure 3a), while in Tajikistan chips had four times more sodium and potassium levels below 2000 mg K/2000 kcal (Figure 3b).

Figure 3b discloses 34 foods that exceeded maximum sodium recommended limits, presenting contents above 2000 and up to 8000 mg Na/2000 kcal. Four of these 34 foods are industrial foods, of which the dry bread crumbs collected in both countries stand out due to their high sodium contents—ranging from 6198 to 7422 mg Na/2000 kcal. Home-made traditional dishes also exceeded the upper recommended limits for sodium—around 4000 mg Na/2000 kcal (*plov*, in Tajikistan) and over 7000 mg Na/2000 kcal (*ashlyamfu*, in Kyrgyzstan). Regarding breads, all types (home-made and industrial) were in the range of 2500 to 5000 mg Na/2000 kcal; and presented potassium contents below or close to 1000 mg K/2000 kcal, with the exception of home-made dark wheat bread (Tajikistan), with 1389 mg K/2000 kcal. Home-made savoury pastries, such as *chebureki*, *piroshky*, *samsa*/*sambusa*, sausage rolls and *belyashi*, are also displayed in this group: sodium content varied between 2000 and 6000 mg Na/2000 kcal and most had potassium levels between 650 and 1000 mg K/2000 kcal. Hot dogs in both countries presented similar levels of sodium (more than 4000 mg Na/2000 kcal) and potassium (more than 1500 mg K/2000 kcal).

Figure 3c shows 17 foods with sodium contents below the upper recommended limits and the lowest potassium contents from all foods collected. Sweet foods predominate and more than half of these foods are industrial, including corn snacks, cookies, and sweet pastries. Home-made sweet pastries, buns, and cakes are also included in this figure. Notably, two sweet foods (e.g., cakes and buns) showed a sodium content per 2000 kcal above 1500 mg.

Table 1 shows the sodium and potassium contents per serving and molar ratios of the most common ready-to-eat predefined food groups, as well as their contributions to the daily recommended limits of these nutrients. In both countries, the main dishes were the principal home-made food source of sodium, contributing almost three-quarters of the maximum sodium recommended limits, followed by home-made sandwiches with a contribution of nearly half of the value. In Tajikistan, home-made breads and industrial snacks had a sodium content representing 31% of the maximum recommended limits each, while in Kyrgyzstan, home-made snacks, bread, and savoury pastries provided 43%, 36% and 33%, respectively, of sodium values with respect to the limits for daily intake. Beverages were shown to be the major sodium source of industrial foods collected in Kyrgyzstan. Main dishes and sandwiches were also the most important sources of potassium in both countries, although their contribution towards the minimum threshold ranged between 9.6% and 22.4%. Home-made snacks presented the highest mean molar Na:K ratio in Tajikistan (21.2), while in Kyrgyzstan industrial beverages had the uppermost molar Na:K ratio (16.4). Home-made buns (Tajikistan) and home-made beverages (Kyrgyzstan) showed the lowest molar ratios from all food groups—1 and 0.1, respectively.

**Figure 4:**
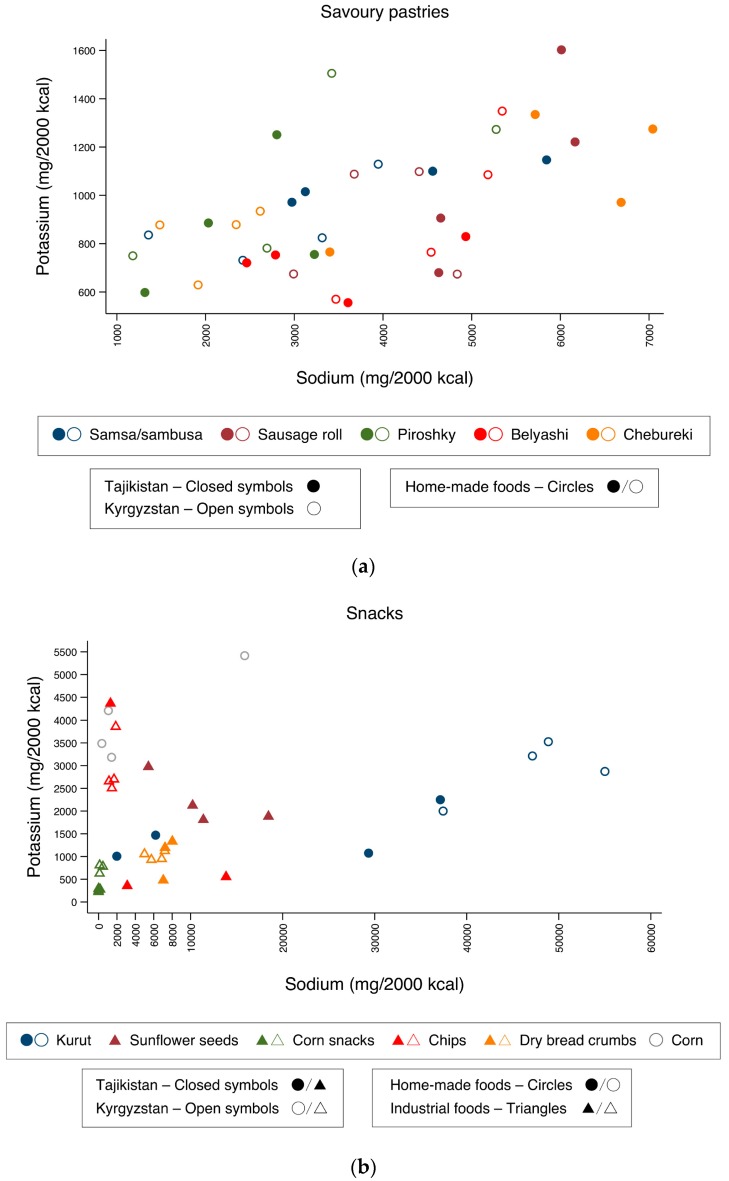

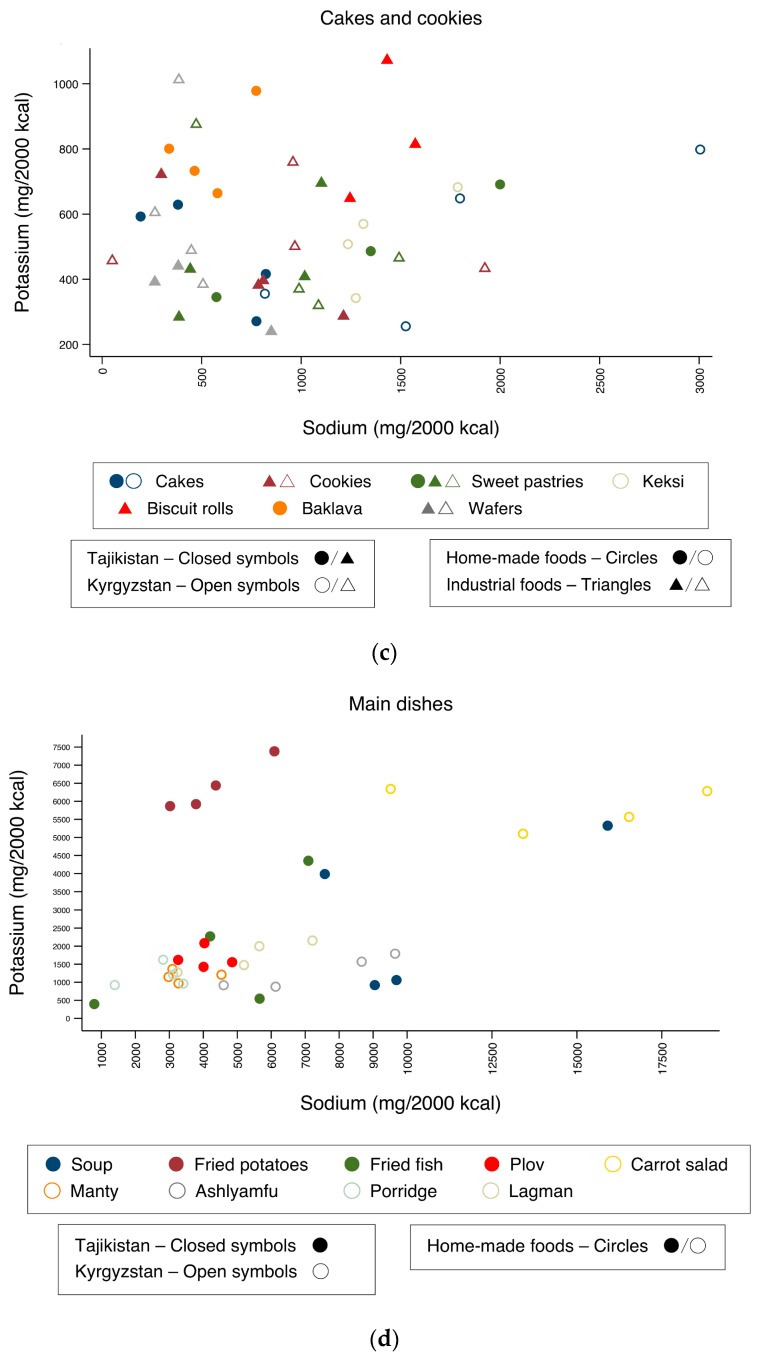

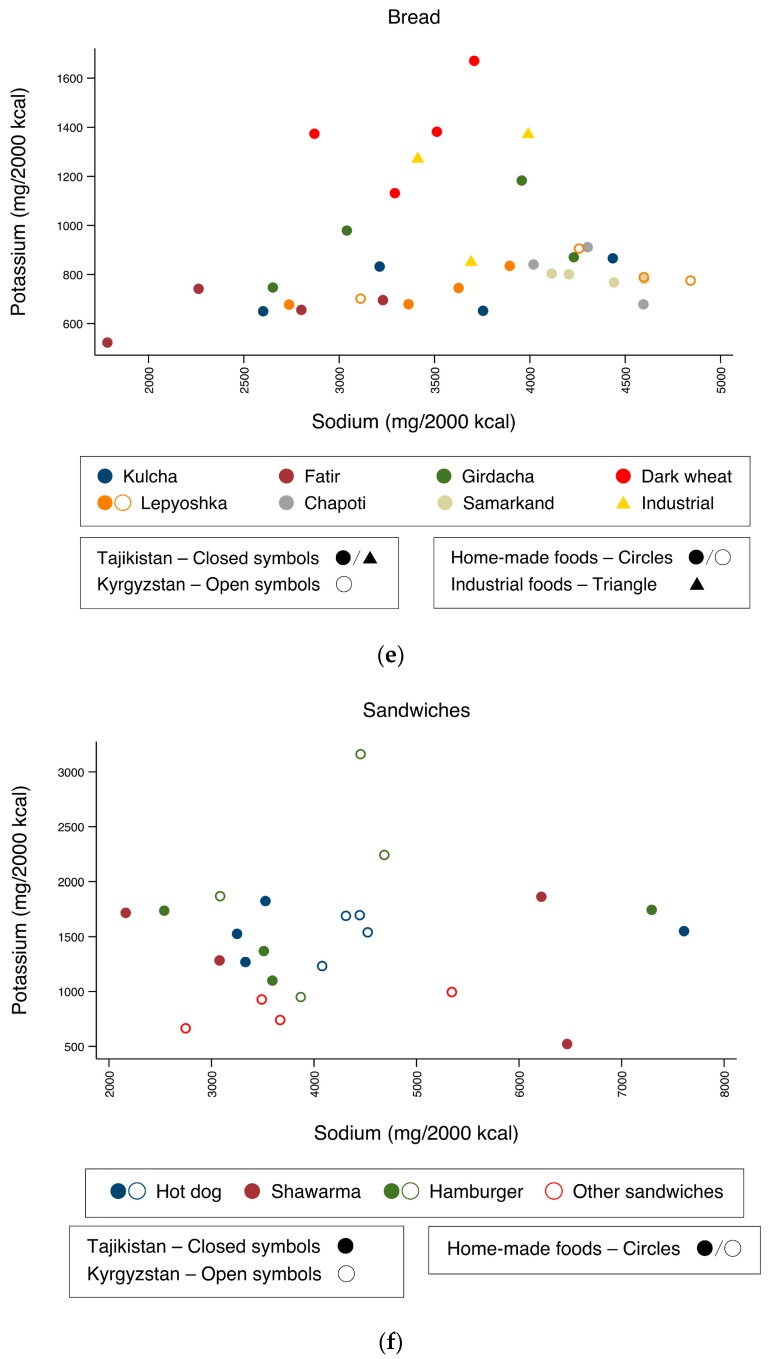
Distribution of individual sodium and potassium contents (mg/2000 kcal) of each of the samples collected for the most commonly available home-made and industrial foods assigned to six of the eight different predefined groups: (**a**) savoury pastries; (**b**) snacks; (**c**) cakes and cookies; (**d**) main dishes; (**e**) bread and (**f**) sandwiches. Closed symbols represent Tajikistan and open symbols represent Kyrgyzstan; circles indicate home-made foods and triangles indicate industrial foods.

Figure 4a–f presents the food groups with the highest average sodium content, disaggregated into all their constituent foods samples and distributed according to individual sodium and potassium contents (in mg/2000 kcal).

In the group of home-made savoury pastries (Figure 4a), the individual samples presented a great variability in their sodium contents, even between the four samples of the same food collected from different vending sites. Most samples had contents above the maximum sodium recommended limits, with some samples reaching up to 5000 mg Na/2000 kcal (Kyrgyzstan) and 7000 mg Na/2000 kcal (Tajikistan).

Regarding snacks (Figure 4b), most samples of home-made *kurut* had extreme sodium contents—between 30,000 and 60,000 mg Na/2000 kcal. In Tajikistan, the majority of chips collected had values above maximum sodium recommended limits, reaching more than 10,000 mg Na/2000 kcal, while in Kyrgyzstan, all chip samples had borderline values with respect to the limits of sodium. In Kyrgyzstan, three out of four samples of corn on the cob had potassium values above 3510 mg K/2000 kcal and sodium content below upper recommended limits, although one of the samples contained almost 20,000 mg Na/2000 kcal.

Within cakes and cookies (Figure 4c), the majority of samples had sodium content below recommendations in both countries, although home-made sweet pastries (Tajikistan) and industrial cookies (Kyrgyzstan) were borderline in terms of the sodium threshold. Exceptionally, one sample of home-made cake (Kyrgyzstan) had a content of 3000 mg Na/2000 kcal.

In the main home-made dishes group (Figure 4d), overall the four samples of each dish were grouped in similar ranges of sodium contents. In samples of carrot salad (Kyrgyzstan) extreme values of between 10,000 mg Na/2000 kcal and more than 17,500 mg Na/2000 kcal were found. In Tajikistan, soups presented the highest sodium contents—between 7000 and more than 15,000 mg Na/2000 kcal; half of them had potassium contents above the minimum potassium recommended limits. Samples of fried potatoes (Tajikistan) had contents ranging between 2000 and around 6000 mg Na/2000 kcal and presented the highest potassium contents, with one of the samples containing approximately 7500 mg K/2000 kcal. Only two samples—porridge (Kyrgyzstan) and fried fish (Tajikistan)—had sodium contents below the upper recommended limits.

Regarding the group of breads (Figure 4e), all samples but one, collected for both countries, had sodium contents above the upper recommended limit, with some of them reaching almost 5000 mg Na/2000 kcal. Most potassium contents varied between 600 and 1000 mg K/2000 kcal. Industrial bread and home-made dark wheat bread samples (both from Tajikistan) presented higher potassium contents in comparison to other bread samples.

Within the samples of home-made sandwiches (Figure 4f), all presented sodium above 2000 mg Na/2000 kcal, with a great dispersion in the contents of this nutrient within the same types of sandwich from the same country. Most had potassium contents between 500 and 2000 mg K/2000 kcal, except two samples of hamburger (Kyrgyzstan) that presented higher levels of potassium.

## 4. Discussion

The results of this study show that street food in Dushanbe and Bishkek can be an important source of dietary sodium and may have a low potassium content and high Na:K ratio, though a large variability is observed across different types of foods or among samples of the same food items obtained from different vendors. As street foods have been shown to be important sources of dietary intake for many people living in LMICs [28], this study is timely and relevant for Tajikistan and Kyrgyzstan since these countries are exploring new ways of preventing NCDs and, in particular, are exploring how to strengthen the promotion of healthy diets [38,39].

In both countries, home-made foods, notably the main traditional dishes, are the leading sources of sodium among street foods, with one serving of these foods contributing, on average, to more than 70% of the maximum daily sodium recommended limits [15]. Other important sources of sodium are home-made sandwiches, snacks, bread, and savoury pastries, in line with previous evidence [40,41].

While in some countries industrial foods are the most important source of dietary sodium [42]; in others, sodium is mainly obtained from the preparation and cooking of foods, which can contribute up to 70–76% of the sodium intake [41,42]. In the case of countries from Central Asia, the high sodium content of traditional foods is suggested to be influenced by the “Silk Road” pattern, in which the tradition of using salt for food preservation strongly remains in the food culture [43]. Additionally, some traditional foods, such as *ashlyamfu* or carrot salads from Kyrgyzstan, contained soy sauce in their recipes, which can also add to the sodium content of the foods [40,41].

Even though home-made foods can be major sources of sodium, main dishes are also significant sources of potassium. For example, despite being sodium-rich, Kyrgyz carrot salad and some Tajik soup samples, including potato and vegetables (sources of potassium [44]), also presented important levels of this nutrient.

Industrial foods were shown to be key sources of sodium as well. The increasing availability of these foods, in the urban contexts of Dushanbe and Bishkek, reflects the nutrition transition that is ongoing in these countries [6,9]. In particular, commonly available industrial beverages from Kyrgyzstan, or industrial snacks from Tajikistan, were shown to largely contribute to the maximum daily sodium recommended limits.

Most food groups presented mean molar Na:K ratios well above the optimal ratio of 1 suggested by the WHO in order to prevent NCDs [19]. This also reaffirms the need for sodium reduction among these foods. The offer of healthy foods, low in sodium and high in potassium, should be encouraged in these settings [28].

In fact, promoting healthy diets should be a priority for Tajikistan and Kyrgyzstan. The prevalence of hypertension in these countries is increasing [45,46] and latest estimates indicate that Central Asia is the region with the highest sodium intake in the world, with a mean of 5.51g/day—almost three times the WHO maximum recommended limit [15]. Unhealthy food environments and diets contributing to a further increase in these sodium intake levels are of major concern [14].

Salt reduction strategies are considered best-buy interventions, as they are effective, feasible, and affordable to implement [2]. Reducing sodium intakes has shown to be beneficial in high-sodium environments, leading to a decrease in BP [47] and directly reducing CVD risk, resulting in long-term impacts on public health [14,42,48].

Different salt reduction strategies have been implemented worldwide, at population or individual levels, including food reformulation, front of pack labelling, regulatory schemes to limit sodium levels in foods, taxation of high-sodium foods, community interventions, and consumer education [49]. Multi-component approaches have been shown to have the most powerful benefits [50]. Combining interventions to engage individuals and relevant stakeholders in health behavior change and population interventions to create healthy food environments could be the best approach in contexts where high sodium contents come from both discretionary salt added while preparing/cooking home-made foods and industrial foods [51].

For home-made foods, one option would be focusing on salt reduction strategies in public education and consumer awareness, as previously done in countries like China and Japan, where, similarly, sodium intake comes typically from salt added during preparation of foods [49]. Particularly, in Tajikistan and Kyrgyzstan, strategies could involve education of street food vendors to both encourage the cooking of healthy local foods and limit the use of discretionary salt or sodium-rich sauces and condiments.

From our findings, the wide ranges of sodium content found with respect to individual samples of some foods show that there is room to cook, prepare, and/or produce foods from all food groups with less added salt [52]. Gradual and small reductions of sodium content in foods have been shown not to affect consumer taste preference, acceptability, and purchase intent [53,54,55,56]. In some cases, it was observed substantial differences even among different samples of the same food, brand and country (e.g., chips from Tajikistan), which reinforces the opportunity to efficiently produce products towards the lower end of the range of sodium content.

In addition, work could be done on increasing consumer education with respect to diet, health, and awareness of the harmful effects of high sodium and low potassium intakes [49,57].

Regarding the sodium content in industrial foods, an option would be that of setting sodium content targets (voluntary or mandatory) for encouraging reformulation. While some countries have set voluntary targets for specific foods, other countries have adopted more comprehensive legislative approaches to set a maximum sodium content of their foods (e.g., South Africa and Argentina) [49]. Setting up maximum limits for food groups that have shown to be major sources of sodium, such as industrial beverages (*maksym* and *chalap*), would be a priority for Kyrgyzstan. Tajikistan could prioritize industrial snacks, such as chips and dry bread crumbs. This approach would require mapping as well as engagement of local food suppliers and further efforts to monitor compliance with regulations or voluntary guidance.

Another approach could be working closely with the industry to improve information available on packaging of industrial foods, as well as educating consumers on label reading. This would entail setting rules for the provision of quantitative ingredient lists, nutrition declarations, and front-of-pack labelling, in order to provide consumers with the necessary information regarding the sodium contents of foods in order to make a decision [49,58]. If companies are required to provide compositional information, it might also serve as a further incentive for them to reduce the sodium in their food.

In addition to sodium-reduction approaches, the overall composition of diets is also of particular importance for determining the impact on CVD outcomes in high-sodium environments [14]. Population-level approaches to concurrently lower sodium and increase potassium intakes may subsequently produce a joint effect in the reduction of BP and CVD risk [17,19,21].

However, the simultaneous compliance with sodium and potassium daily recommendations may be a challenge which needs to be supported by the promotion of healthy and affordable eating patterns [59]. Diets, such as the Mediterranean and the Dietary Approaches to Stop Hypertension (DASH) diets—rich in fruits, vegetables, legumes, nuts, whole-grains, seafood and vegetable oils, with moderate consumption of dairy products and less red meat, processed foods, and added fats and sugars—have shown to be associated with lower BP and to improve cardiometabolic health and other chronic diseases [60,61,62,63,64]. With adjustments to locally available foods and taking into account cultural preferences, the dietary recommendations of these diets could be translated to the context of Tajikistan and Kyrgyzstan to help promote healthy and accessible diets, without increasing dietary costs in these settings [7].

For example, fruits and vegetables, which are low in sodium and high in potassium, were observed to be widely available in the markets of Dushanbe and Bishkek, despite the fact that their daily consumption continues to decline in these countries [10]. Their adequate consumption is associated with a lower risk of mortality, notably from CVDs, and could be promoted as a key component of a healthy diet in these countries [65].

Likewise, nuts and legumes, also linked to better cardiometabolic outcomes [66], were frequently found in all markets visited during the study and could also be promoted, rather than certain sources of animal protein such as red meat and, especially, processed meat [7].

Furthermore, dietary guidance could also be focused on sodium–potassium ratios [59]. Advice could be given based on the food groups with the highest Na:K ratios, which could include recommendations to moderate the consumption of industrial snacks and beverages as well as home-made snacks, which may greatly contribute to high sodium intakes. Boiled corn in the cob, commonly found in all markets, may be a healthy alternative snack due to its high potassium content, especially when no salt is added before consumption. The variability of sodium content in corn may be most dependent upon the quantity of salt commonly added by the vendor before selling or upon customer request. One particular sample may have been exceptionally salted in excess (i.e., purposely, to increase the flavor or added twice, by mistake), which reinforces the need for education strategies targeting the use of discretionary salt and consumer awareness about the risks of its excess consumption. In addition, seasonality may condition fruits and vegetables’ availability and is an important element to take into account for dietary guidance.

To our knowledge this is the first study that provides data on the nutritional composition of ready-to-eat street foods in Central Asia. The study was carefully designed to provide an accurate and representative assessment of the nutritional content of these foods from Dushanbe and Bishkek. The number of different samples collected for each food, from different vending sites, gives us important insights into the ranges of sodium and potassium content, which may be helpful when planning interventions. However, the availability of street foods may not necessarily reflect the usual dietary intake of the urban population in both countries, although it is expected that the contribution of street food to total food consumption is high in these settings [28]. Results of this study also cannot be generalized to other settings (e.g., rural areas), where the availability and the nutritional composition of foods may differ. Other important sodium-rich and potassium-poor food sources sold in settings distinct from markets and their surroundings may have been missed.

In this sense, the study provides useful information on the quantity of sodium and potassium in commonly available foods and may be used as a starting point to promote dietary changes to help the population in Tajikistan and Kyrgyzstan to achieve recommendations for the intake of these nutrients. Nevertheless, it does not replace data on dietary intake nor does it provide information on the overall quality of diets in these countries.

Monitoring the impact of interventions on intake levels is crucial when implementing successful national strategies related to these nutrients [67]. Available methods include 24-h urine collection, spot urine collection and dietary surveys. Using 24-h urine collection is considered the gold standard method for establishing sodium baseline intake, for both individuals and populations [67]. Potassium baseline intake may also be accurately measured through 24-h urine excretion and 24-h dietary recall methods [68]. However, using 24-h urine collection method for monitoring and evaluation can pose a high burden for surveillance in LMICs, as it requires full capacity and financial and political support [69]. A less expensive, practical, and accurate monitoring approach, at an individual level, may include assessing repeated casual urine Na/K ratios, which may help to understand the features of high, intermediate and low Na/K ratio individuals [68]. Urinary Na/K ratios have shown to be a more precise index for tracking the contribution of changes in sodium and potassium intakes not only to BP but also to CVD risk, when compared to measuring the levels of these nutrients separately [70]. However, none of these methods substitute the identification and monitoring of changes in the nutritional composition of food sources.

Likewise, it is essential to assess baseline sodium and potassium contents in other important sources of these nutrients and to systematically monitor the impact of interventions on the nutritional composition of foods. Further evaluation may focus on food categories that present the highest average sodium contents and Na:K ratios, and for which specific targets should be set beforehand [71]. Alternatively, for industrial prepacked foods, analyzing both food labelling and sales data, may help identifying further sources and tracking the ones contributing the most to sodium exposure as a result of their high sodium content and high consumption by the population [72]. Nevertheless, this would require the main food retailers to be engaged in the reformulation process and willing to share data and accurate updates to the nutrient declarations of their products. In both countries, additional efforts aiming at commonly imported products are also needed.

## 5. Conclusions

In summary, promoting the nutritional quality of street foods should be a priority to be integrated into wider work on nutrition and food security in Tajikistan and Kyrgyzstan. The large variability observed in this study across different types of foods and similar products acquired in different vending places translates the possible patterns of consumption of street food, as well as shows the large potential for improvement of the street food environment by promoting the healthiest foods available. The creation of healthier food and drink environments and the regular monitoring of specific targets set for key food sources will contribute towards the delivery of national action plans on NCDs and the achievement of the WHO’s voluntary global targets of a 30% reduction in mean population sodium intake and a 25% reduction in risk of CVDs by 2025 [73].

## Figures and Tables

**Table 1:** Sodium (Na) and potassium (K) content and molar Na:K ratio in different groups of the most commonly available street foods in Tajikistan and Kyrgyzstan.

		Mean Serving Size (g) *	Na				K				Molar Na:K Ratio		
			Mean (Min–Max) * mg/Serving			% Recom. ^1^	Mean (Min–Max) * mg/Serving			% Recom. ^2^	Mean (Min–Max) *		
***Tajikistan***													
**Industrial foods**	*N* *												
Bread	3	50.0	240	234	243	12.0	75	56	87	2.1	5.6	4.6	7.4
Cakes and cookies	14	59.1	103	30	238	5.2	66	21	133	1.9	3.1	0.7	7.2
Snacks	14	38.6	621	0	2218	31.0	129	17	447	3.7	10.2	0.0	42.6
**Home-made foods**	*N* *												
Bread	27	122.4	620	325	839	31.0	152	95	279	4.3	7.3	3.5	11.5
Bun	4	60.0	50	0	97	2.5	68	51	94	1.9	1.0	0.0	2.1
Cakes and cookies	11	88.2	112	45	275	5.6	105	31	169	3.0	2.4	0.6	4.9
Main dishes	16	361.4	1485	109	3724	74.2	788	55	1922	22.4	5.5	0.9	17.6
Sandwiches	12	222.4	962	442	1588	48.1	336	128	632	9.6	6.0	2.1	21.1
Snacks	4	18.0	559	50	1325	28.0	44	24	80	1.3	21.2	3.3	46.4
Savoury pastries	20	75.1	364	158	619	18.2	85	37	123	2.4	7.4	3.7	11.7
***Kyrgyzstan***													
**Industrial foods**	*N* *												
Beverages	8	200.0	579	432	986	29.0	67	42	115	1.9	16.4	8.8	29.4
Bun	4	45.5	127	5	381	6.3	47	37	65	1.3	3.8	0.2	10.0
Cakes and cookies	12	50.3	76	4	139	3.8	65	17	144	1.9	2.9	0.2	7.5
Snacks	12	31.6	183	9	528	9.2	104	25	244	3.0	4.0	0.2	12.3
**Home-made foods**	*N* *												
Beverages	4	200.0	4	0	15	0.2	82	13	122	2.3	0.1	0.0	0.2
Bread	4	120.0	720	521	867	36.0	135	117	149	3.9	9.0	7.5	10.6
Bun	4	66.2	132	115	157	6.6	91	63	116	2.6	2.6	1.8	3.5
Cakes and cookies	8	122.4	347	124	590	17.3	113	33	180	3.2	5.5	3.9	10.1
Main dishes	20	402.7	1409	241	2639	70.5	438	98	856	12.5	5.7	2.5	11.9
Sandwiches	12	222.1	1078	443	1858	53.9	423	107	1187	12.1	5.5	2.4	9.1
Snacks	8	150.5	861	66	1848	43.0	321	52	645	9.1	14.9	0.2	32.5
Savoury pastries	20	136.1	661	141	1424	33.0	178	90	407	5.1	6.3	2.7	12.2

^1^ World Health Organization (WHO) recommends sodium intake of less than 2000 mg/day [15]. ^2^ World Health Organization (WHO) recommends potassium intake of at least 3510 mg/day [18]. * Mean, minimum and maximum values take into account sodium or potassium contents (in mg/serving) or molar Na:K ratios of individual samples included in each food group. *N* values correspond to the number of individual samples included in each food group.
